# A Case of Colon Cancer and Pauci-Immune Crescentic Glomerulonephritis

**DOI:** 10.7759/cureus.27460

**Published:** 2022-07-29

**Authors:** David Wilhelm, Dawn Caster, Susan Coventry, Gunjan Garg

**Affiliations:** 1 Internal Medicine, University of Louisville, Louisville, USA; 2 Nephrology, University of Louisville, Louisville, USA; 3 Pathology, Norton Children's Hospital, Louisville, USA

**Keywords:** anca-negative, dialysis, acute kidney injury, invasive colon cancer, pauci-immune crescentic glomerulonephritis

## Abstract

The association between membranous nephropathy and cancer has been well documented. Crescentic glomerulonephritis (GN) has also been associated with various types of cancers. To our knowledge, there has only been one previously documented case of seronegative pauci-immune crescentic glomerulonephritis associated with colon cancer. We present a case of a 51-year-old male with newly diagnosed high-grade poorly-differentiated colon carcinoma who was found to have seronegative pauci-immune crescentic GN with 70% involvement of glomeruli on renal biopsy. The patient was treated with pulse steroids and rituximab with resolution of acute kidney injury.

## Introduction

The most common glomerular pathology in patients with solid malignant tumors is membranous nephropathy [1]. Crescentic glomerulonephritis (GN) has been associated with several solid tumor malignancies, typically associated with renal cell, gastric, and lung cancers. Crescentic GN (also referred to as rapidly progressive GN) is a life-threatening disease which is characterized by rapid loss of kidney function clinically and crescentic formation pathologically [2]. Crescentic GN is classified into three main categories, including anti-glomerular basement membrane (anti-GBM) crescentic GN, immune-complex crescentic GN, and pauci-immune crescentic GN. Pauci originates from the Latin word "paucus", meaning little or few, and pauci-immune indicates the almost complete absence of immunoglobulin deposits [3]. A majority of pauci-immune crescentic GN cases are antineutrophil cytoplasmic antibody (ANCA) positive, including those associated with malignancies [4]. To our knowledge, there has only been one previously reported case of seronegative pauci-immune crescentic GN associated with colon cancer.

## Case presentation

**Figure 1:**
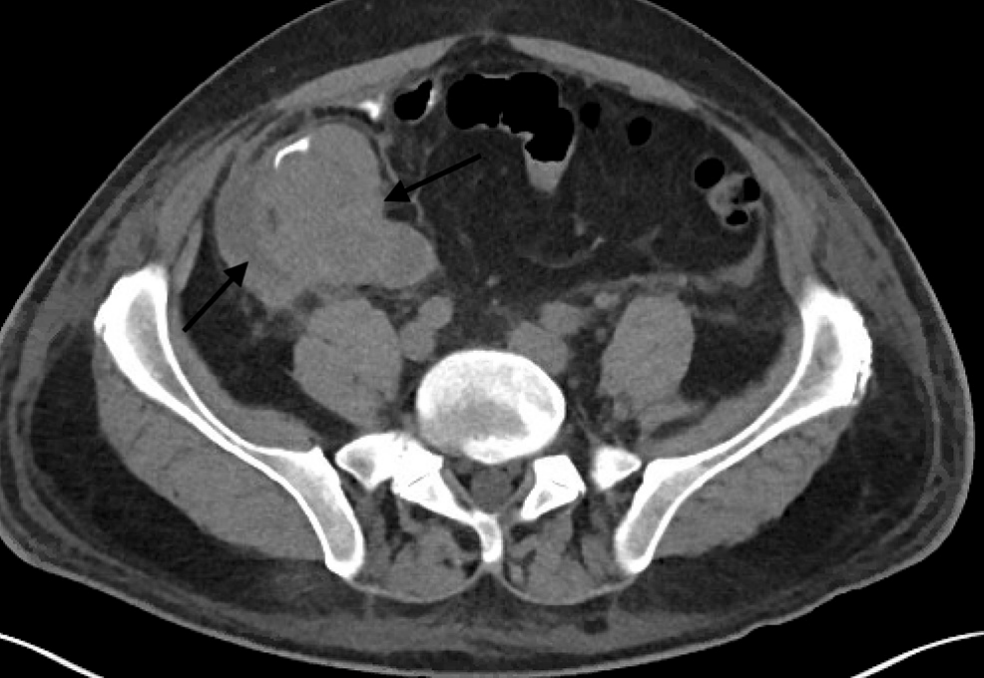
Large heterogeneous soft tissue mass identified within the right lower quadrant, which indents upon the ascending colon with significant mass-like thickening of the cecum (black arrows)

**Figure 2:**
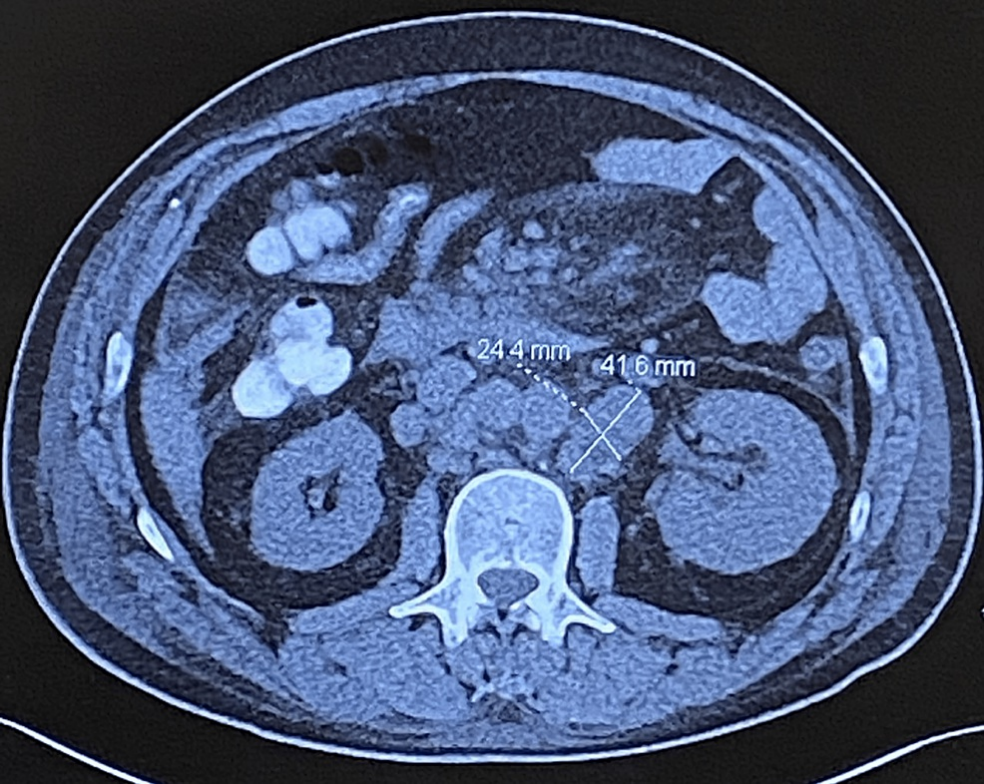
Left periaortic retroperitoneal node measuring 4.2 cm x 2.4 cm

A 51-year-old male presented with complaints of abdominal pain, nausea, vomiting, and 25-pound weight loss over six weeks. CT of the abdomen/pelvis revealed a mass in the right lower quadrant with significant mass-like thickening of the cecum and significant diffuse upper abdominal, retroperitoneal, and mesenteric lymphadenopathy (Figures 1-2). Colonoscopy showed a large infiltrative mass in the cecum measuring 6-7 cm extending to the ileocecal valve. Biopsy was consistent with high-grade colon carcinoma. A staging CT of the chest was performed and showed multiple enlarged mediastinal lymph nodes.

Serum creatinine on admission was 5.1 mg/dL, with the previous creatinine being 1.8 mg/dL one month prior. The baseline creatinine was unknown. The patient endorsed a longstanding history of microscopic hematuria when routinely examined for work physicals, with episodic gross hematuria that was preceded by upper respiratory symptoms. He denied any other history concerning for intrinsic renal disease. On admission, urinalysis showed 50+ red blood cells, 30-49 white blood cells, trace leukocyte esterase, and trace bacteria. Specific gravity was 1.016. The urine microalbumin to creatinine ratio (UMA/Cr) was 930 mcg/mg, and the urine protein to creatinine ratio (UPCR) was 2.1 g/g. The renal ultrasound was unremarkable. Serologies, including antinuclear antibodies, ANCA, and anti-GBM antibodies, were negative, and complement levels (C3, C4) were normal. Serum creatinine peaked to 5.4 mg/dL on admission day two and began down trending with IV fluid resuscitation. The patient was eventually discharged after one week in the hospital with plans for outpatient follow-up. Serum creatinine on discharge was 4.2 mg/dL. Renal biopsy was considered but was held off as the renal function was improving appropriately with conservative management.

**Figure 3:**
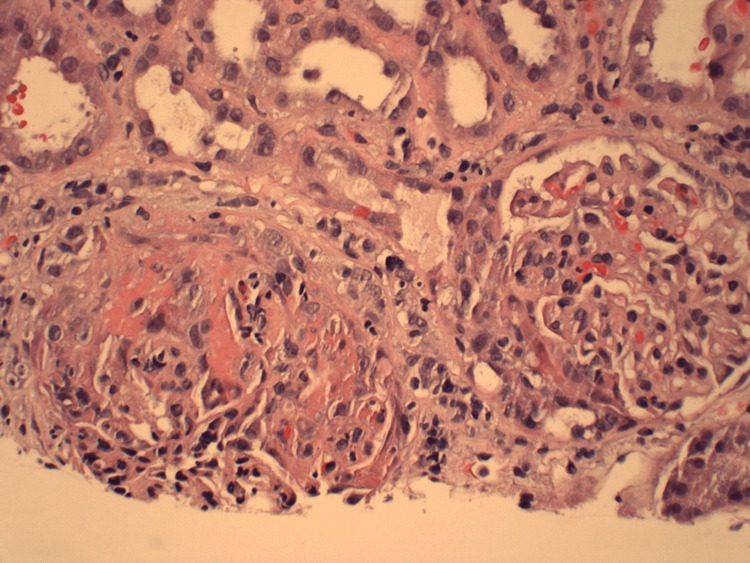
H&E stain on light microscopy of renal biopsy showing crescentic glomerulonephritis

**Figure 4:**
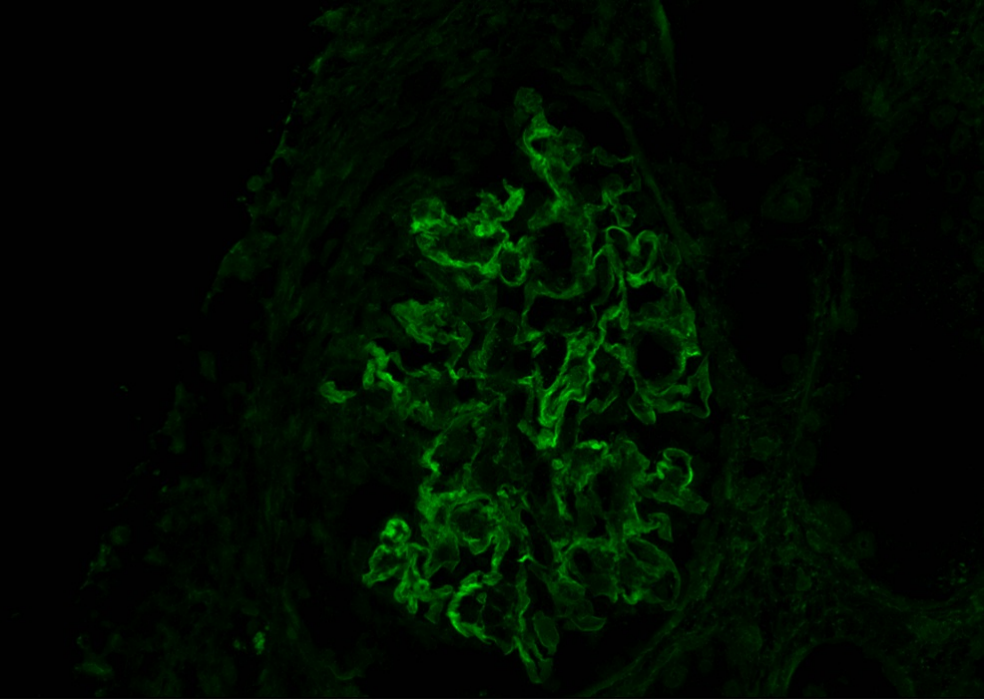
Immunofluorescence showing 2+ IgG linear staining on capillary loops

**Figure 5:**
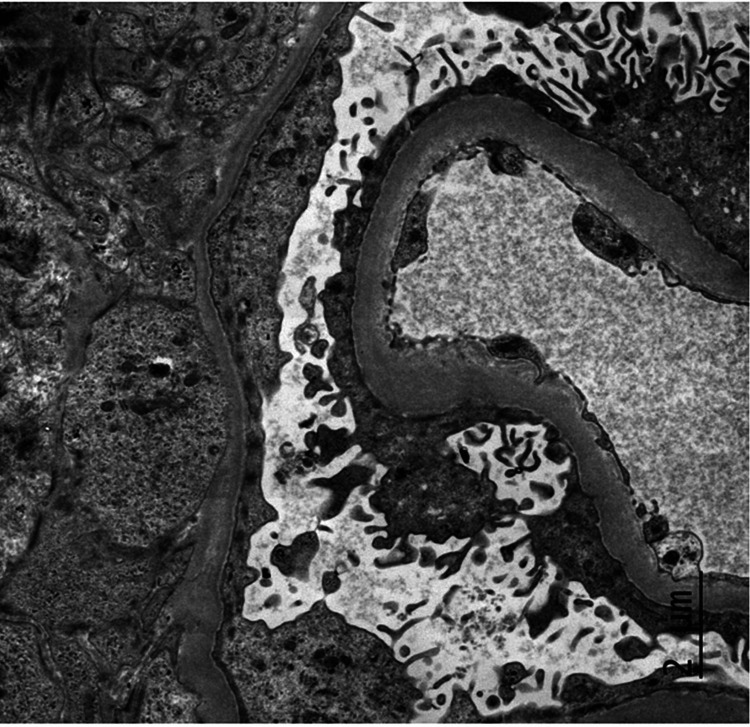
Electron microscopy showing reactive podocytes with widespread but incomplete foot process effacement

Four days following hospital discharge, the patient followed up in the oncology clinic with plans for initiating cancer treatment. A comprehensive metabolic panel obtained at that visit showed serum creatinine elevated to 5.9 mg/dL. The patient was readmitted to the hospital. Serum creatinine on the day of admission was 6.4 mg/dL. On day two of this hospital admission, dialysis was initiated due to uremia with blood urea nitrogen of 87 accompanied by jerking movements and oliguria with volume overload. Serologies were repeated and were again negative. Renal biopsy was pursued due to worsening kidney function, and light microscopy showed crescentic GN with 70% of the glomeruli involved (Figure 3). Immunofluorescence (IF) showed 2+ diffuse linear capillary loop staining for IgG (Figure 4) and 1+ staining for IgA, kappa, and lambda. IgM and C1q were negative. There was 4+ diffuse granular mesangial C3 and 3+ wispy peripheral fibrinogen in seven out of 10 glomeruli consistent with crescents. Because serum anti-GBM Ab was negative twice, the IF finding of linear IgG staining was considered nonspecific. Electron microscopy showed no granular electron-dense deposits or paraprotein deposits but did show reactive podocytes with widespread foot process effacement (Figure 5). Basement membranes were normal in thickness overall. Treatment was started with methylprednisolone 1 gm daily for three days, followed by prednisone 60 mg daily, with plans to taper on outpatient follow-up. Rituximab 1 gm was given on days one and 14.

During this admission, serum creatinine peaked to 7.6 mg/dL. Dialysis continued until day nine of treatment, with seven dialysis sessions completed in total, and the patient was discharged on day 15 of treatment with a down trending serum creatinine to 3.6 mg/dL. Acute kidney injury (AKI) resolved by day 25 with a serum creatinine of 1.7 mg/dL.

## Discussion

To our knowledge, this is only the second documented case of seronegative pauci-immune crescentic GN associated with colon cancer. As mentioned previously, a majority of pauci-immune crescentic GN cases are ANCA positive, including those associated with malignancies. Glomerular diseases associated with malignancies are not believed to be directly related to tumor burden but are believed to be caused by tumor cell products such as hormones, growth factors, cytokines, and tumor antigens [5]. Lionaki et al. delved deeper into the mechanism of pauci-immune crescentic GN secondary to malignancy in ANCA-positive patients and proposed several mechanisms, including the effect of tumor-associated antigens, antibodies, and products reacting with the capillary walls, inducing inflammation, direct effects of tumor cells on the endothelium, potential of lymphocyte activation and induction of monoclonal immunoglobulin activity, and the formation of antibodies directed toward endothelial antigens. They state that ANCAs are pathogenic and that resting neutrophils that have ANCA autoantigens can be induced by tumor-related factors, causing damage to endothelial cells [5].

The pathogenesis of pauci-immune crescentic GN secondary to malignancy in ANCA-negative patients is less clear. Villacorta et al. state that much like in the case of ANCA-positive pauci-immune crescentic GN, neutrophils are also thought to be major effector cells in ANCA-negative cases [6]. They also suggest that neutrophil degranulation could be induced by antibodies other than ANCA, such as anti-endothelial cell antibodies (AECA) or autoantibodies to human lysosomal membrane protein-2 (LAMP-2).

The previously reported case of seronegative pauci-immune crescentic GN associated with colon cancer was published in 2014 in the Indian Journal of Nephrology [7]. Per the case report, the 46-year-old female patient had a history of colon carcinoma managed with chemotherapy and partial colectomy. She was admitted with a creatinine elevation to 3.1 mg/dL and had negative serological tests prior to undergoing a renal biopsy, which showed crescentic GN. Treatment was initiated with steroids and cyclophosphamide, and creatinine on discharge was 2.4 mg/dL.

Another case presentation highlighted a 74-year-old male who was diagnosed with poorly differentiated rectal adenocarcinoma that was treated with a combination of surgical resection and six cycles of capecitabine chemotherapy who was diagnosed with an acute kidney injury and underwent a renal biopsy that showed pauci-immune crescentic GN [8]. Unfortunately, in this case, the patient had worsening renal function in addition to worsening hepatic function due to metastatic disease and passed away after he elected for palliative care.

In a recent case series, 74 patients with ANCA-negative pauci-immune crescentic GN diagnosed between August 2006 and December 2018 were included [9]. Among the 74 patients included in the case series, which is believed to be the largest reported series of ANCA-negative pauci-immune crescentic GN to date, six cases were associated with malignancy - two cases of lung cancer, one pharyngeal cancer, one laryngeal cancer, one chronic lymphocytic leukemia (CLL), and one lymphoma.

## Conclusions

In summary, we present a case of seronegative pauci-immune crescentic GN associated with colon cancer, which to our knowledge, is only the second documented case. There have, however, been reported cases of seronegative pauci-immune crescentic GN associated with other types of malignancies, even though this has been reported less frequently than cases in ANCA-positive patients.

In the case of our patient, the differential diagnosis was initially broad. Given the patient's history of microscopic hematuria on routine work physicals with episodic gross hematuria, there were initial concerns about IgA nephropathy. Biopsy more definitively led us to the correct diagnosis and, therefore, proper treatment. Subsequently, the patient had rapid improvement in kidney function and was able to come off dialysis to pursue cancer treatment. The patient had one follow-up in the nephrology clinic 11 days after hospital discharge, with creatinine returning to the previous value of 1.7 mg/dL. However, he, unfortunately, passed away four weeks later due to metastatic cancer.

## References

[1] Jhaveri KD, Shah HH, Calderon K, Campenot ES, Radhakrishnan J (2013). Glomerular diseases seen with cancer and chemotherapy: a narrative review. Kidney Int.

[2] Chen YX, Chen N (2013). Pathogenesis of rapidly progressive glomerulonephritis: what do we learn?. Contrib Nephrol.

[3] Rutgers A, Sanders JS, Stegeman CA, Kallenberg CG (2010). Pauci-immune necrotizing glomerulonephritis. Rheum Dis Clin North Am.

[4] von Vietinghoff S, Schneider W, Luft FC, Kettritz R (2006). Crescentic glomerulonephritis and malignancy - guilty or guilt by association?. Nephrol Dial Transplant.

[5] Lionaki S, Marinaki S, Panagiotellis K (2020). Glomerular diseases associated with malignancies: histopathological pattern and association with circulating autoantibodies. Antibodies.

[6] Villacorta J, Diaz-Crespo F, Acevedo M, Guerrero C, Mollejo M, Fernandez-Juarez G (2016). Antineutrophil cytoplasmic antibody negative pauci-immune extracapillary glomerulonephritis. Nephrology.

[7] Kar S, Rathod S, Abreu E, Kar P (2014). A case of paraneoplastic syndrome associated rapidly progressing glomerulonephritis in a patient with colon cancer. Indian J Nephrol.

[8] Swift O, Ramanarayanan S, Paterson A, Mathavakkannan S (2021). Pauci-immune crescentic glomerulonephritis associated with metastatic rectal carcinoma. J R Coll Physicians Edinb.

[9] Ronsin C, Georges M, Chapelet-Debout A (2022). Anca-negative pauci-immune necrotizing glomerulonephritis: A case series and a new clinical classification. Am J Kidney Dis.

